# The 90 90 90 strategy to end the HIV Pandemic by 2030: Can the supply chain handle it?

**DOI:** 10.7448/IAS.19.1.20917

**Published:** 2016-06-30

**Authors:** David Jamieson, Scott E Kellerman

**Affiliations:** Partnership for Supply Chain Management (PFSCM), Arlington, VA, USA

**Keywords:** HIV/AIDS, supply chain management, 90-90-90, PEPFAR, commodities

## Abstract

**Introduction:**

UNAIDS “90-90-90” strategy calls for 90% of HIV-infected individuals to be diagnosed by 2020, 90% of whom will be on anti-retroviral therapy (ART) and 90% of whom will achieve sustained virologic suppression. Reaching these targets by 2020 will reduce the HIV epidemic to a low-level endemic disease by 2030. However, moving the global response towards this universal test and treat model will pose huge challenges to public health systems in resource-limited settings, including global and local supply chain systems. These challenges are especially acute in Africa, which accounts for over 70% of the persons affected by HIV.

**Discussion:**

From a supply chain perspective, each of the “90's” has possible complications and roadblocks towards realizing the promise envisioned by 90-90-90. For instance, ensuring that 90% of HIV-infected persons know their status will require a large increase in access to HIV tests compared with what is currently available. To ensure that there are enough anti-retrovirals available to treat the nearly 25 million people that will require them by 2020 represents a near doubling of the ARV supplied to treat the 13 million currently on treatment. Similarly, to monitor those on treatment means an unprecedented scale-up of viral load testing throughout Africa.

**Conclusions:**

Larger issues include whether the capacity exists at the local level to handle these commodities when they arrive in the most severely affected countries, including considerations of the human resources and costs needed to make this strategy effective. We believe that such “real world” analysis of proposed strategies and policies is essential to ensure their most effective implementation.

## Introduction

In 2014, UNAIDS announced bold new targets for the global response to HIV, aptly named the 90-90-90 strategy, that 90% of people living with HIV (PLHIV) know their status, 90% of diagnosed PLHIV are on treatment and 90% of PLHIV on treatment achieve an undetectable viral load, by 2020 [1]. The global supply chain system, from producer through to patient, ensures adequate stocks of drugs and associated commodities necessary to achieve targets for each of the “90s.” This system, already stretched, will require a critical rethinking in order to meet these ambitious targets. While not an issue of supply, there is likely enough production capacity in the world to actually make the drugs and commodities required. Rather, challenges come farther down the supply chain, into the countries where there may not be the capacity to handle the increased volume down to the last leg of the supply chain or last-mile delivery where the supply chain infrastructure is often less efficient and costly.

Each “90” poses specific challenges due largely to already strained infrastructure, but also opportunities for innovation, particularly in sub-Saharan Africa which accounts for over 70% of persons living with HIV. As regards the first 90, at most 50% of infected persons currently know their status, globally, despite few strains in the global supply chain for testing commodities, and many community actors keen to bring testing to their neighbours [2]. The second 90 poses the greatest challenge with estimates that, to support over 28 million on treatment in sub-Saharan Africa alone, means delivering 30 containers of medicines across Africa each day, every day (Iain Barton, personal communication). Lessons to tackle this huge logistical challenge in the most affected countries can be taken from commercial sector supply chains. The final 90 is already changing with efforts to bring viral load (VL) monitoring to countries that currently rely on a symptom-driven approach or CD4 testing to gauge the treatment progress [3]. Here, we offer a review of challenges and potential solutions to strengthen supply chains and deliver on the promise of 90-90-90.

## Discussion

### 90% of infected persons know their status

Unless 90% of people living with HIV know their status, the 90-90-90 strategy will not succeed, but current UNAIDS estimates suggest only 50% of those infected are diagnosed, and 70% of those tested are women, most often during prenatal care [2]. More opportunities are needed to diagnose men. Generalized screening campaigns (e.g. national testing days) raise awareness, but are less effective for identifying infected people. Drawing on recent evidence from Kenya, the United States Global AIDS Coordinator, Dr. Deborah Birx reports some sites have not identified an HIV-infected person in two years [4], potentially resulting in some sites with expiring stocks of rapid diagnostic tests (RDTs), while sites with higher numbers of newly diagnosed patients lack the RDTs to serve their populations.

As PEPFAR pivots to targeted testing strategies, doubling the number diagnosed will not require simply doubling the number of tests performed. By focusing on the highest prevalence areas and those at greatest risk, we estimate that a 50% increase in the volume of tests is required to achieve 90% of infected persons knowing their status. Although the global supply market has the capacity to meet this need, a risk is reliance on the Determine test, which had an 83% market share in 2014 despite 12 other HIV RDTs that are prequalified by the World Health Organization (WHO) [5]. Should a catastrophic event impact the manufacture of Determine, targets will be put at risk. This risk could be mitigated by expanding procurement to alternative, equally effective tests and revisiting testing algorithms, which have remained static for years.

The 2015 WHO HIV testing guidelines recommend strategies that will impact in-country RDT supply chains, including substantial increases in testing by community lay providers and increases in home-based and self-testing in the coming years. The private sector (for-profit and voluntary or not-for-profit) may hold an untapped potential to expand testing services in currently unconventional settings, for example, home or community testing, the workplace, major transport arteries or free testing within private clinics.

Each of these approaches would require strengthening the last-mile delivery in the final leg of the supply chain to the point of service, which is generally the most difficult and costly segment of the supply chain. To mitigate the risk of stock-outs due to unpredictable demand, larger stocks may need to be held at service delivery points and intermediary points along the supply chain. Resupply strategies should also become more flexible with more frequent deliveries responding to consumption, rather than one or two bulk deliveries each year. This brings proven commercial sector strategies for consumable items (e.g. supermarket goods) into the health sector.

### 90% of HIV+ persons are on treatment

Full implementation of 90-90-90 requires a near doubling of the demand for ART [6] from 15.8 million person-years (PYR) in mid-2015 to 24 million PYR by 2018 [7], and nearly 30 million PYR by 2020. This increase will strain many parts of already congested health systems and will require disciplined planning and close collaboration within and between countries to avoid overwhelming the systems.

More than 80% of all donor-funded ARVs purchased since 2006 were supplied by Indian generic manufacturers [8]. These suppliers regularly expanded capacity as coverage increased from less than a million a decade ago to today's approximately 15 million people on treatment, and they must grow again to meet the new WHO guidelines to treat all [9]. The response to market signals for large increases in ARV demand will require clear forecasting data for manufacturers, an understanding of the speed of increase in patients treated and confidence that sustainable funding will be available. With these assurances in place, manufacturers can be expected to invest, provided they see an adequate rate of return.

Global collaboration will be essential and the role of the WHO in collating national plans, targets and treatment statistics will be crucial. WHO holds an annual meeting with ARV manufacturers that will take on increased importance as 90-90-90 expands, providing an independent forum to discuss funding, demand and supply [10].

**Figure 1:**
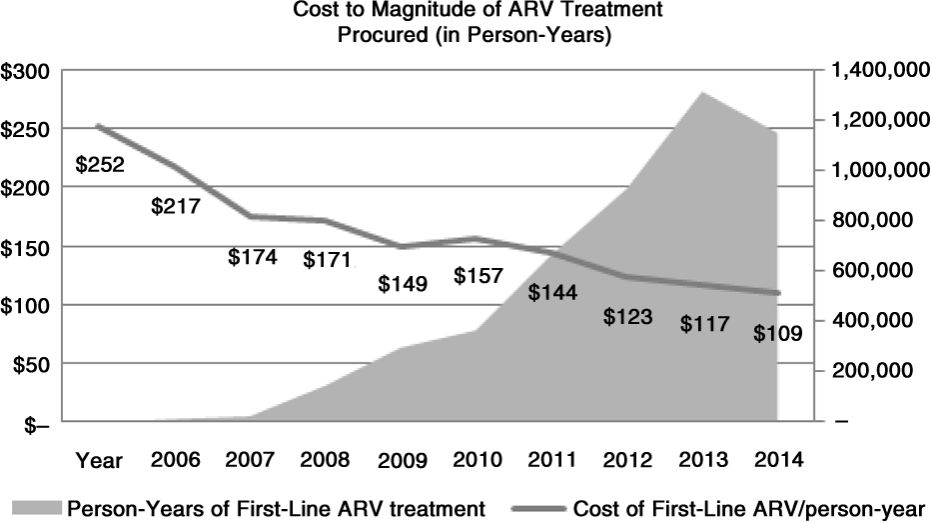
Decrease in price of first-line ARV treatment, 2006 to 2014. First line ARV regimens included in this table: Efavirenz/Emtricitabine/Tenofovir 600/200/300 mg [Atripla], tablets, 30 Tabs Efavirenz/Emtricitabine/Tenofovir 600/200/300 mg, tablets, 30 Tabs Efavirenz/Lamivudine/Tenofovir 600/300/300 mg, tablets, 30 Tabs Lamivudine/Nevirapine/Zidovudine 150/200/300 mg, tablets, 60 Tabs Lamivudine/Zidovudine/Efavirenz 150/300/600 mg, tablets, co-blister 60+30 Tabs Cost is aggregate cost/person-year of all regimens From Partnership for Supply Chain Management (PFSCM),un published analysis of procurement data, 2006 to 2014.

Treatment costs could be a major challenge to reaching targets. Although ART costs have declined dramatically over the last decade, due primarily to the increasing reliance on generic formulations (Figure 1), further innovation is essential [11]. Most of the current first-line generic drugs are at or close to their optimum level for a sustainable market, as demonstrated by the price trends reported by WHO [12]. The next leap forward in treatment cost reductions will come from new drugs at lower dosages. The active pharmaceutical ingredient (API) in ARVs accounts for 80% or more of the total cost. By reducing the amount of API, the next generation of ARVs promises to be significantly cheaper [13]. In aggregate, these reductions may be partially offset by increased demand for more expensive second and third drug regimens as VL monitoring identifies treatment failure.

A major challenge is to bring these new, cheaper and often better drugs to developing countries quickly. Historically, it has taken more than five years for generic versions of newly patented drugs to become widely available at affordable prices. This period is shortening, but improved coordination, innovation and planning are needed to close the gap. Actions that currently happen in series need to happen in parallel, such as the initial registration by companies that develop these drugs with a stringent regulatory authority or WHO pre-qualification, WHO treatment guidance, the licensing of generic manufacture via the Medicines Patent Pool and, ultimately, in-country registrations, approval and production. This requires a more deliberative planning process and closer collaboration between manufacturers, regulators and programs, including pooled procurement to provide the volume needed when a product is launched.

It is essential that the current arrangements under the World Trade Organization Trade-Related Aspects of Intellectual Property Rights (TRIPS) mechanism continue, by which generic versions of new drugs under patent in developed countries are made available in low and middle income countries, and new legal barriers are not created.

The impact of increased volumes and new product introduction will require innovation and some risk-taking if country supply chains are not to collapse under the weight of needed commodities. Stock-outs of ARVs and commodities have long plagued HIV programs in sub-Saharan Africa and elsewhere with important clinical consequences [14,15]. Storage and distribution systems need a radical redesign to adapt to the high throughput of increased volumes and pivot to a more focused approach concentrating on key populations and high prevalence areas. Although governments must continue to direct policy and country-level strategy, meeting 90-90-90 demand will require a total market approach incorporating supply, storage and distribution services from public, voluntary and the for-profit private sectors to release significant, untapped capacity outside government systems.

### 90% of HIV+ persons on treatment achieve viral suppression

At present, the numbers of CD4 and VL tests are far below the level one would expect from WHO treatment guidelines. In sub-Saharan Africa, less than half of persons living with HIV infection have access to VL testing [16], and only two of seven countries reported more than 40% of patients receiving one or more VL tests [17]. The good news is that the major manufacturers of VL equipment have anticipated the need to monitor 30+ million patients per annum and are bringing new models to market with the requisite manufacturing capacity. The challenge is to extend accredited laboratories to ensure high-quality results, referral networks and distribution systems to meet the need at the patient level. Currently, there are no point-of-care VL machines on the market, although two may be released in 2016 (the OMNI Cephied and Alere Q). In the meantime, the key logistical challenge is to build strong referral networks to get blood samples or dried blood spots to labs, and data networks that can promptly return results.

Network optimization is essential to place VL machines in the right place to match the focus on key populations and high prevalence areas. Analysis of CD4 capacity shows that placing machines, including point-of-care equipment, in all areas has often led to major over-capacity, significantly increasing the cost per test [18].

## Cross-cutting themes

Internationally, there is more than adequate capacity to transport these commodities from their ports of origin to their destination. The use of sea freight offers significant savings compared to air freight, but requires good advance planning due to longer transit times, which can cut into shelf life, and to account for peak or holiday periods when demand is higher and ports may be closed.

Integrated planning is essential to achieving 90-90-90. From a supply chain perspective, the three 90s are often managed separately at the local level, particularly when estimating demand, for example, testing targets in many countries are not always linked to treatment goals, which are not reflected in laboratory monitoring and compromise accurate forecasting for each.

Human resource constraints present additional challenges, as they do in most of the health systems in which HIV/AIDS is prevalent. A priority is to address the challenges involved in maintaining ARVs through an efficient procurement and supply chain management system by establishing a dedicated procurement team or at the least providing a cadre of well-trained specialist supply chain staff [19].

Untapped potential exists in the private sector. Private laboratories can be contracted on a service model and paid on a per test basis, saving governments the capital cost of equipment, while using contract terms to ensure quality-assured standards. Health insurance schemes being introduced by several countries may be particularly interested in optimizing use of the private sector in order to drive value.

## Conclusions

The scientific advances, political drive and advocacy behind 90-90-90 make an end to AIDS, unthinkable only a few years ago, a realistic goal. Close cooperation between policymakers, operational planners and supply chain personnel is essential to manage the rapid scale-up to reach 90-90-90 targets. With determination, commitment and careful planning, the supply chain will be a key enabler. Science or political will alone will not determine success; well-thought-out strategies developed by public health professionals with inputs from industry, nongovernmental organizations and the commercial sector are needed to provide the leadership that will enable 90-90-90 to succeed.
